# Endothelial p16 expression identifies a subgroup of clear cell renal cell carcinoma with favorable prognosis

**DOI:** 10.1186/s12885-026-17178-6

**Published:** 2026-10-26

**Authors:** Michael Stoffel, Katharina Baschun, Melina Kehl, Jörg Ellinger, Niklas Klümper, Sebastian Kadzik, Franziska I. Winterhagen, Manuel Ritter, Michael Hölzel, Glen Kristiansen, Marieta I. Toma

**Affiliations:** 1 Institute of Pathology, University Hospital Bonn, Bonn, Germany; 2 Department of Urology, St. Josef-Hospital, Troisdorf, Germany; 3 Department of Urology and Pediatric Urology, University Hospital Bonn, Bonn, Germany; 4 Center of Integrated Oncology – CIO ABCD, University Hospital Bonn, Bonn, Germany; 5 Institute of Experimental Oncology, University Hospital Bonn, Bonn, Germany; 6 Department of Urology, Johanniter-Krankenhaus Bonn, Bonn, Germany

**Keywords:** Clear cell renal cell carcinoma, Endothelial cells, Vessels, p16, Senescence, Survival

## Abstract

**Background:**

Clear cell renal cell carcinoma (ccRCC) is characterized by extensive angiogenesis and marked architectural heterogeneity. While vascular density has been investigated as a prognostic parameter in ccRCC, little is known about the biological characteristics of intratumoral endothelial cells.

**Methods:**

In this study, we analyzed the p16 expression in a tissue microarray cohort comprising 347 ccRCC cases with clinicopathological and survival data and related the vascular p16 expression to previously described tumor architecture subtypes.

**Results:**

Tumor architecture could be assessed in 315 cases and was classified into nine predominant growth patterns. Tumors with thick trabecular/insular or solid sheet architecture were grouped as high-risk patterns and were associated with higher tumor grade and shorter overall survival (*p* < 0.001). Tumor cell p16 expression was infrequent and showed no association with clinicopathological parameters or survival. In contrast, endothelial p16 expression was commonly observed in intratumoral blood vessels, with a median of 35% positive vessels per case. Higher endothelial p16 expression, both in extent and intensity, was significantly associated with lower tumor grade, lower pT stage, favorable architectural patterns, and prolonged overall survival. Furthermore, the prognostic impact of architectural growth patterns was most pronounced in tumors with low endothelial p16 expression, whereas tumors with high endothelial p16 expression showed comparable survival independent of architectural subtype.

**Conclusion:**

Endothelial p16 expression is common in ccRCC and is associated with favorable clinicopathological characteristics and prolonged survival. These findings suggest that endothelial p16 expression may complement architectural tumor stratification in ccRCC, identifying cases with good prognosis.

## Introduction

Clear cell renal cell carcinoma (ccRCC) accounts for approximately 80% of all renal cell carcinomas and represents the most common malignant tumor of the kidney [1, 2]. Despite advances in surgical management and systemic therapy, ccRCC remains associated with substantial morbidity and mortality worldwide [3]. A hallmark of ccRCC biology is its pronounced angiogenesis, largely driven by inactivation of the von Hippel–Lindau (VHL) tumor suppressor gene and subsequent activation of hypoxia-inducible factor (HIF)-dependent signaling pathways, particularly the VEGF axis [4–6]. This highly vascular phenotype is not only a defining biological feature of ccRCC but also forms the basis for modern anti-angiogenic therapeutic strategies [7, 8].

Beyond conventional clinicopathological parameters such as stage, grade, and necrosis, ccRCC exhibits remarkable morphological and biological heterogeneity [9]. Recent studies demonstrated that ccRCC can be subdivided into distinct architectural growth patterns with different prognostic and molecular characteristics [10, 11]. In particular, compact solid and thick trabecular growth patterns have been associated with more aggressive tumor behavior, while nested and microcystic architectures tend to correlate with more favorable outcomes. These observations suggest a close relationship between tumor architecture, vascular organization, and tumor biology. Distinct vascular architectural patterns, including pseudoacinar, lacunar, and scattered vascular arrangements, emphasize the heterogeneity of the ccRCC vasculature [12].

While angiogenesis in ccRCC has been extensively investigated, considerably less is known about the biological characteristics and functional states of intratumoral endothelial cells. Tumor-associated endothelial cells are increasingly recognized as active components of the tumor microenvironment rather than passive structural elements. Beyond regulating oxygen and nutrient supply, endothelial cells contribute to immune regulation, vascular permeability, stromal remodeling, and therapeutic response. Endothelial heterogeneity may therefore represent an additional layer of biological complexity in ccRCC progression [13].

In this context, cellular senescence has emerged as an important biological program in both tumor cells and the tumor microenvironment. Cellular senescence is characterized by a stable cell-cycle arrest induced by various forms of cellular stress, including oncogenic signaling, DNA damage, oxidative stress, or replicative exhaustion. Among the major regulators of senescence, the p16INK4a-Rb pathway plays a central role [14, 15]. p16 expression has been widely used as a senescence-associated marker in different physiological and pathological settings, although its biological significance may vary depending on tissue context [16, 17]. In endothelial cells, senescence-associated phenotypes have been implicated in chronic vascular and renal diseases and may influence inflammatory signaling, vascular remodeling, and tissue homeostasis. However, the role of endothelial p16 expression and putative vascular senescence in ccRCC remains poorly understood [18].

In the present study, we analyzed p16 expression in tumor cells and intratumoral endothelial cells in a large cohort of ccRCC and correlated these findings with architectural growth patterns, clinicopathological parameters, and patient survival. Our aim was to investigate whether endothelial p16 expression identifies biologically and clinically distinct vascular phenotypes with potential prognostic relevance in ccRCC.

## Materials and methods

### Patient cohort

Archived formalin-fixed, paraffin-embedded tissue samples from 347 patients who underwent radical or partial nephrectomy for clear cell renal cell carcinoma (ccRCC) between 2006 and 2015 at the University Hospital Bonn were included in this study. The median patient age was 66 years (range, 17–89 years). Tumors were staged according to the TNM Classification of Malignant Tumors (2020 edition), and grading was performed according to the ISUP grading system [19]. Clinicopathological data are summarized in Table 1. Follow-up data were obtained from the institutional clinical registry and were available for 248 patients. The median follow-up time was 92 months (range, 1–199 months).

**Table 1:** Description of our TMA cohort and its characteristics: sex, grading, TNM-Stage

Parameter	Category	Number
Age	Median (range)	66 (17–89)
Sex	Female	118
	Male	229
pT stage	Organ confined (pT1/2)	240
	Organ non-confined (pT3/4)	107
pN status	pN0/cN0	327
	pN1	20
Grading (ISUP)	G1/G2	282
	G3/G4	65
Distant metastases	M0	310
	M1	37
Total no. of patients		347

Due to differences in tissue availability and evaluability, denominators differed between analyses. Tumor cell p16 expression was assessed in 347 cases. CD31 staining was also assessed in 347 cases. Quantitative vascular p16 expression could be examined in 325 cases. Tumor growth patterns were assessable in 315 cases after exclusion of cases without an assignable dominant pattern. Survival analyses were performed using the respective subset of cases with available follow-up data and evaluable variables.

The study was approved by the Ethics Committee of the University Hospital Bonn (EK 233/20).

### Tissue microarray construction

Tissue microarrays (TMAs) were constructed using the Minicore3 system (Alphelys, Plaisir, France) and the 01-MIN-P10 Punch-Set (Mitogen). For each case, two morphologically representative tumor cores with a diameter of 1 mm were selected. This two-core approach was chosen to improve tissue representation, while recognizing the inherent limitation that TMA-based analyses assess selected tumor areas and may not capture the full extent of intratumoral heterogeneity.

### Immunohistochemical staining for p16

Immunohistochemical staining for p16 was performed using the Ventana ultraView DAB detection system (Roche, Basel, Switzerland). Briefly, 2 μm-thick formalin-fixed, paraffin-embedded tissue sections were mounted on TOMO slides and automatically deparaffinized using the BenchMark Ultra platform (Roche, Basel, Switzerland). Antigen retrieval was performed using Cell Conditioning Solution 1 (CC1, pH 8) for 64 min at 95 °C, followed by endogenous peroxidase blocking with hydrogen peroxide.

The primary antibody against p16 (clone E6H4, Roche, Basel, Switzerland) was applied according to the manufacturer’s protocol. Nuclei were counterstained with hematoxylin. Slides were subsequently dehydrated using graded alcohols and xylene and coverslipped using Pertex mounting medium. Finally, slides were digitally scanned using a Panoramic Midi scanner (3DHISTECH, Budapest, Hungary) and analyzed microscopically.

### Immunohistochemical staining for CD31

To validate the vascular nature of the assessed structures, immunohistochemical staining for CD31 was performed using a semiautomatic staining protocol on the Autostainer 480 S (Epredia, Portsmouth, UK). Briefly, 2 μm-thick formalin-fixed, paraffin-embedded tissue sections were mounted on TOMO slides and dried at 65 °C for 30 min. Deparaffinization and antigen retrieval were performed using the PT module and citrate buffer pH 6.0 (Medac, Wedel, Germany) for 20 min at 99 °C. The primary antibody against CD31 (Agilent, Santa Clara, USA; clone JC70A; dilution 1:250) was applied for 30 min. Detection was performed using an HRP/DAB-based detection system. Nuclei were counterstained with hematoxylin, and slides were dehydrated using graded alcohols and xylene and coverslipped using Pertex mounting medium.

### Microscopic assessment of vascular growth patterns

Hematoxylin and eosin (H&E)-stained sections were used to evaluate tumor architecture. Tumors were classified according to the histomorphological growth patterns described by Cai et al., [10] The predominant architectural pattern was determined across all available cores from each case.

Small and large nest patterns were characterized by tumor cell clusters of varying size surrounded by a prominent capillary network. Thick trabecular/insular patterns showed broader trabecular arrangements separated by fibrous stromal bands. Tumors with compact architecture and reduced vascularization were categorized as solid sheet pattern. Microcystic tumors exhibited cystic spaces, frequently exceeding 100 μm in diameter, whereas tubular/acinar patterns displayed gland-like architectural features.

**Fig. 1:**
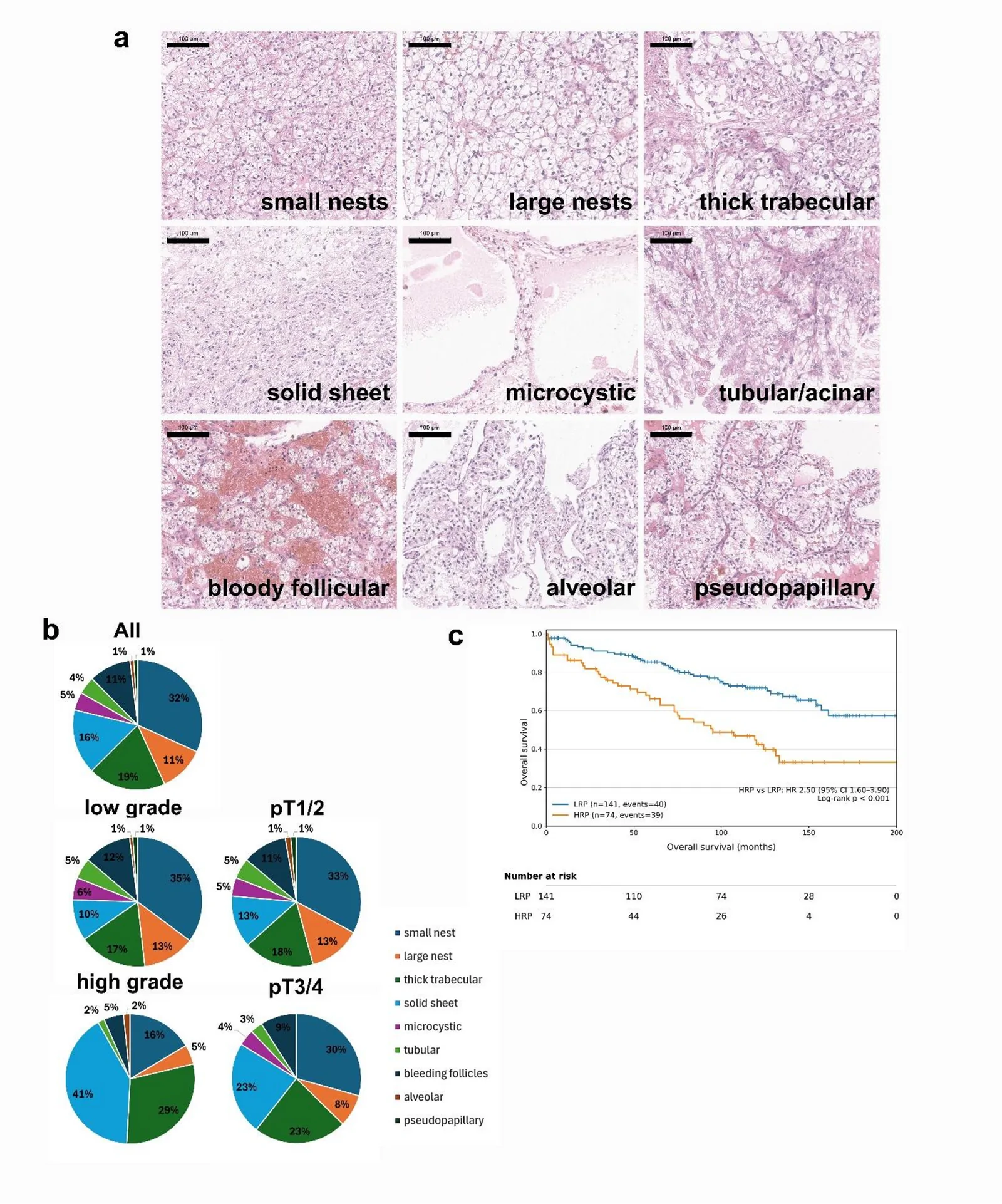
Growth patterns with clinic-pathological parameters and overall survival.** a** Examples of the nine assessed architectural growth patterns: small nests, large nests, thick trabecular/insular, solid sheet, microcystic, tubular/acinar, bloody follicular, alveolar, and papillary/pseudopapillary. **b** Distribution of architectural growth patterns overall and stratified by tumor grade and pT stage. Low grade was defined as ISUP grade 1/2 and high grade as ISUP grade 3/4. **c** Kaplan–Meier analysis of overall survival comparing low-risk patterns (LRP) and high-risk patterns (HRP). HRP included thick trabecular/insular and solid sheet patterns; all remaining patterns were classified as LRP

The “bloody follicular pattern” was characterized by blood-filled spaces with prominent erythrocyte accumulation, often readily identifiable at low magnification. Alveolar patterns resembled compact or nested architectures with smaller cystic spaces within tumor nests. Papillary/pseudopapillary patterns showed tumor cells arranged along papillary structures (Fig. 1).

### Microscopic evaluation of p16 staining

For each tissue core, cytoplasmic p16 immunoreactivity in endothelial cells was assessed microscopically. The percentage and staining intensity of p16-positive endothelial cells were recorded using a predefined and consistently applied scoring approach. When present, p16 expression in tumor cells was additionally recorded. Staining intensity was categorized as negative, low, moderate, or high. For statistical analysis, the highest staining intensity observed per case was used. The proportion of p16-positive endothelial cells was estimated in 10% increments and averaged across all available cores from each case for further analysis.

### Statistical analysis

Statistical analyses were performed using SPSS version 29 (IBM Corp., Armonk, NY, USA). Differences between groups were analyzed using the Mann–Whitney U test. Correlation analyses were performed using Pearson correlation analysis. Survival analyses were conducted using Kaplan–Meier curves and the log-rank test. P-values < 0.05 were considered statistically significant.

Cox proportional hazards regression analyses were performed to assess associations with overall survival. Univariable Cox regression analyses were performed separately for clinicopathological variables, architectural growth pattern, and vascular p16 expression. A final multivariable Cox regression model included pT stage, tumor grade, nodal status, distant metastases, architectural growth pattern, and the proportion of p16-positive vessels. Hazard ratios (HRs) with 95% confidence intervals (CIs) were reported. Cox regression analyses were performed as complete-case analyses using the respective cases with available overall survival data, event status, and evaluable variables included in the respective model. The proportional hazards assumption was assessed using scaled Schoenfeld residuals.

## Results

### Growth patterns are associated with tumor biology and survival

Architectural growth patterns according to the classification proposed by Cai et al., could be assessed in 315 cases [10]. The most prevalent growth pattern in our cohort was the small nest pattern (31.7%), followed by the thick trabecular/insular pattern (19.4%) and the solid sheet pattern (16.2%; 51/315 cases). All remaining growth patterns occurred less frequently (Fig. 1).

Survival analysis revealed that tumors with thick trabecular/insular and solid sheet architecture showed less favorable clinical outcomes compared with the remaining growth patterns. Based on these findings, these two architectural subtypes were grouped as high-risk patterns (HRP), whereas all remaining patterns were categorized as low-risk patterns (LRP). Patients with HRP tumors showed significantly shorter overall survival compared with patients with LRP tumors (log-rank test, *p* < 0.001).

With regard to clinicopathological parameters, high-risk growth patterns were significantly associated with high tumor grade (G3/G4) and the presence of distant metastases compared with low-risk patterns (Mann–Whitney U test, *p* < 0.001 and *p* = 0.038, respectively). No significant associations were observed between growth pattern category and tumor stage or lymph node status.

### Endothelial p16 expression is common in ccRCC vasculature

We next analyzed p16 expression in tumor cells and intratumoral endothelial cells in the ccRCC cohort. Tumor cell p16 expression was infrequent, with only 60 of 347 tumors (17.3%) showing focal clusters of p16-positive tumor cells. Tumor cell p16 expression was not associated with any clinicopathological parameter.

**Fig. 2:**
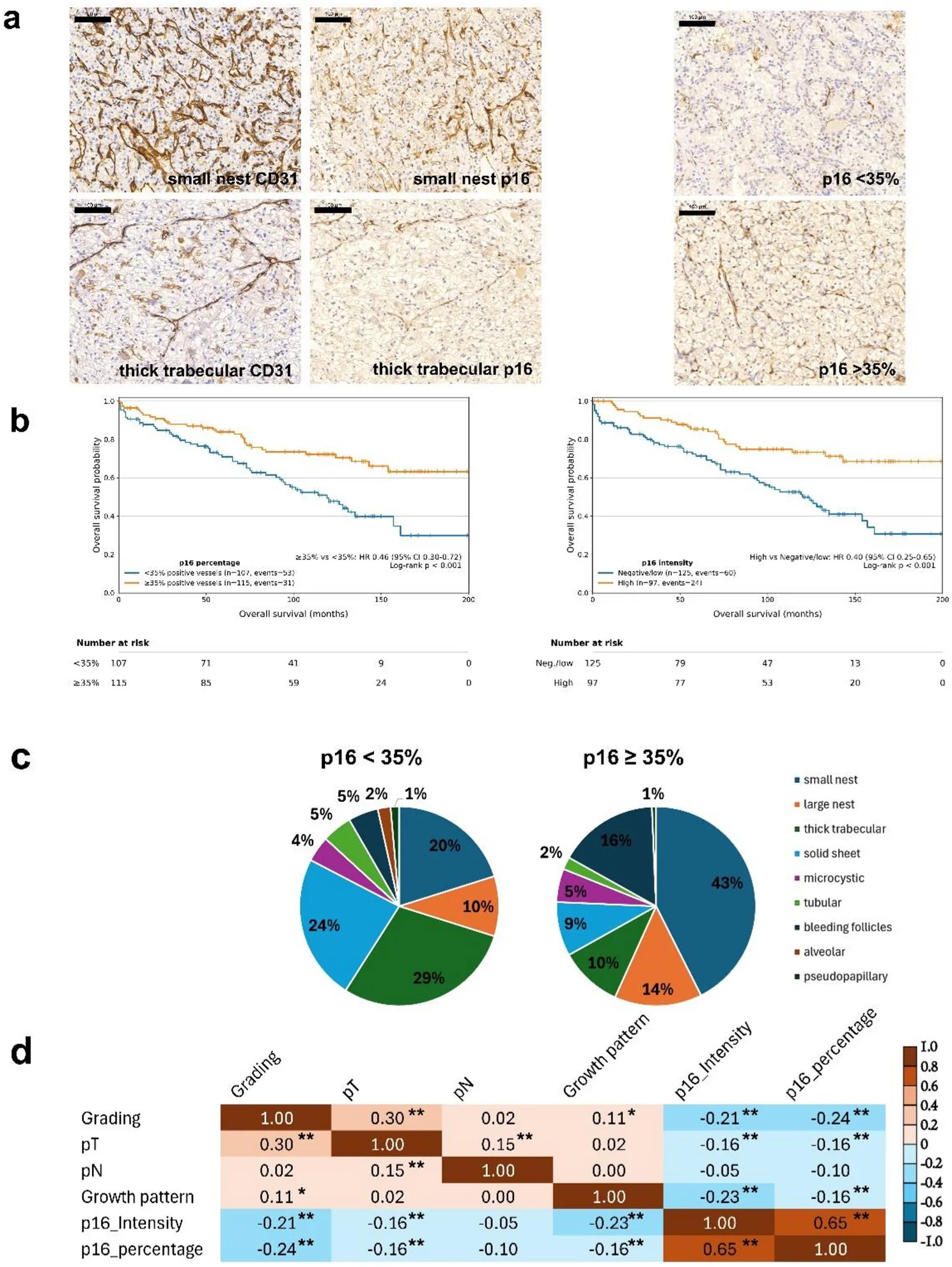
Endothelial p16 expression in ccRCC with clinicopathological parameters and survival.** a** Representative CD31 and p16 staining of small nest and thick trabecular growth patterns with low (< 35%) and high (≥ 35%) vascular p16 expression. **b** Kaplan-Meier analysis of overall survival according to the proportion of p16 positive endothelial cells and p16 expression. **c** Distribution of growth patterns according to p16 vessel expression (**d**) Correlation matrix of clinicopathological parameters, growth patterns and p16 expression

In contrast, p16 expression was frequently observed in intratumoral blood vessels and was present in 285 out of 325 (87.7%) cases. The proportion of p16-positive vessels varied considerably between tumors, ranging from 0% to 100%, with a median of 35% positive vessels per case. Endothelial staining intensity ranged from low to high (Fig. 2a). The side by side comparison of CD31 positive vessels and p16 staining confirmed, that the endothelial cells are p16 positive.

### Vascular p16 expression is associated with favorable clinicopathological parameters and prolonged survival in ccRCC

Higher endothelial p16 expression was significantly associated with favorable clinicopathological features. Both the proportion of p16-positive vessels and staining intensity were significantly higher in low-grade tumors (G1/G2) compared with high-grade tumors (G3/G4) (Mann–Whitney U test, both *p* < 0.001). Similarly, organ-confined tumors (pT1/pT2) showed significantly higher endothelial p16 expression and staining intensity than locally advanced tumors (pT3/pT4) (*p* = 0.001 and *p* = 0.004, respectively).

Tumors with lymph node metastases exhibited significantly lower endothelial p16 positivity and staining intensity compared with node-negative tumors (*p* = 0.014 and *p* = 0.002, respectively). Likewise, cases with distant metastases showed reduced endothelial p16 expression compared with non-metastatic tumors (*p* = 0.007 and *p* = 0.01, respectively).

Kaplan–Meier survival analysis demonstrated that tumors with high endothelial p16 expression, defined either by strong staining intensity or by a proportion of p16-positive vessels above the cohort median of 35%, were associated with significantly prolonged overall survival compared with tumors showing low or absent endothelial p16 expression (log-rank test, both *p* < 0.001; Fig. 2b). Notably, cases with low endothelial p16 staining intensity showed survival comparable to p16-negative cases.

### Vascular p16 expression correlates with growth patterns and tumor grade in ccRCC

Tumors with small nest architecture showed high endothelial p16 expression, both in staining intensity and in the proportion of positive vessels, whereas solid sheet and thick trabecular/insular growth patterns exhibited lower endothelial p16 positivity (Fig. 2c).

Pearson correlation analysis further demonstrated significant associations between clinicopathological parameters, growth patterns, and endothelial p16 expression. Higher pT stage correlated positively with higher tumor grade (*p* < 0.001) and the presence of lymph node metastases (*p* = 0.009). In contrast, higher pT stage was inversely associated with endothelial p16 staining intensity and the proportion of p16-positive vessels (both *p* = 0.004). No significant association was observed between pT stage and tumor growth pattern category.

Both endothelial p16 staining intensity and the proportion of p16-positive vessels were significantly associated with lower tumor grade and more favorable architectural growth patterns (Fig. 2d). In addition, staining intensity strongly correlated with the proportion of p16-positive vessels. Collectively, these findings indicate that increased endothelial p16 expression is associated with a less aggressive tumor phenotype in ccRCC.

### Prognostic impact of architectural growth patterns according to endothelial p16 expression in ccRCC

Because endothelial p16 expression and architectural growth patterns were significantly associated, we next analyzed overall survival according to both parameters. Among tumors with absent or low endothelial p16 expression, high-risk growth patterns were associated with significantly shorter overall survival compared with low-risk growth patterns (log-rank test, *p* = 0.009).

**Fig. 3:**
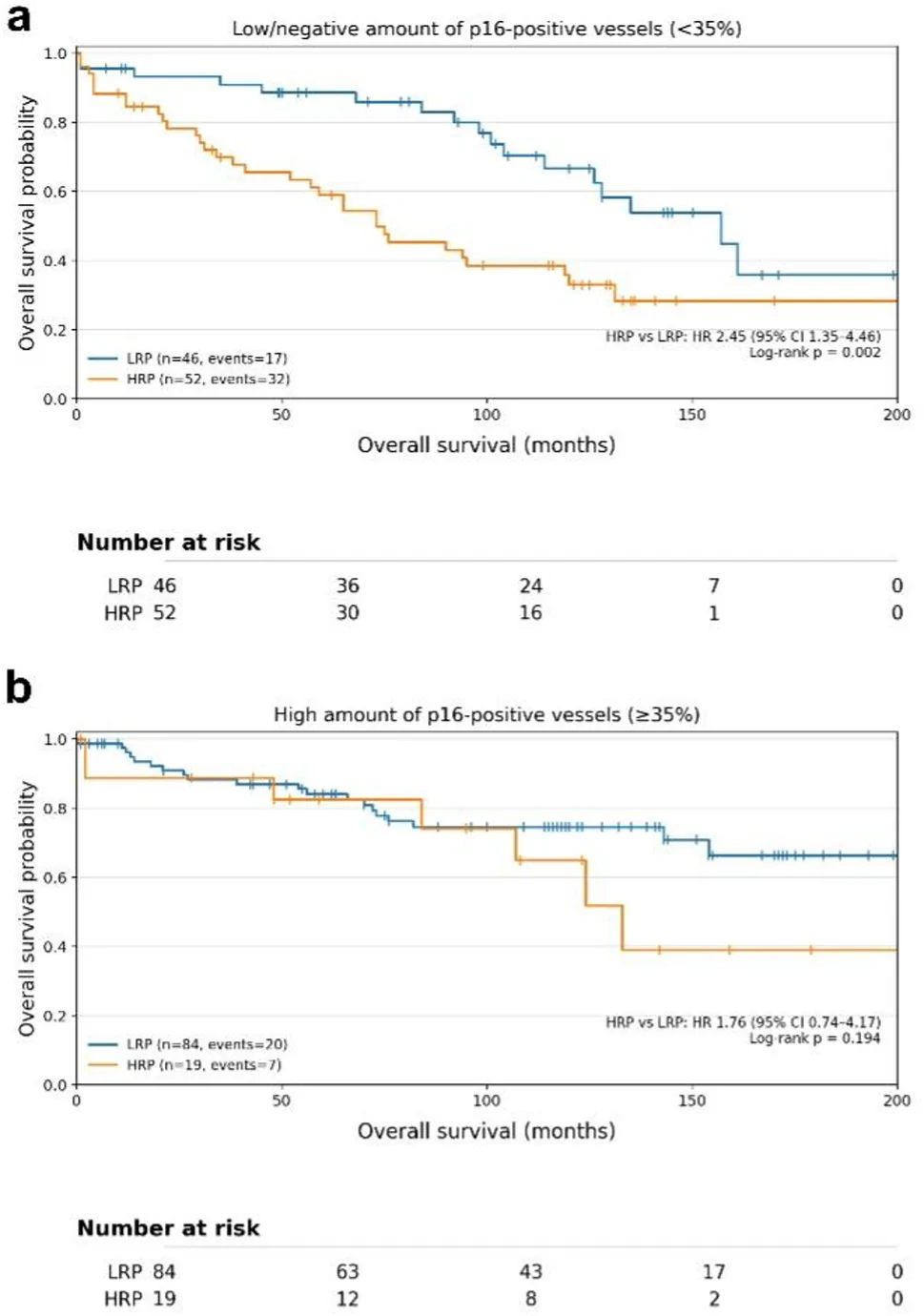
Prognostic impact of growth patterns stratified by p16 expression. (**a**) Kaplan-Meier analysis of overall survival comparing LRP and HRP tumors among cases with low/negative vascular p16 expression. (**b**) Kaplan-Meier analysis of overall survival comparing LRP and HRP tumors among cases with high vascular p16 expression

In contrast, among tumors with high endothelial p16 staining intensity, no significant survival differences were observed between high-risk and low-risk growth patterns. Similar findings were obtained when stratifying tumors according to the proportion of p16-positive vessels. In tumors with low endothelial p16 positivity, low-risk growth patterns showed significantly better overall survival than high-risk growth patterns (log-rank test, *p* = 0.002). However, in tumors with ≥ 35% p16-positive endothelial cells, survival differences between high-risk and low-risk growth patterns were no longer significant (log-rank test, *p* = 0.194). The prognostic impact of growth patterns stratified by endothelial p16 expression is shown in Fig. 3.

These findings support a close relationship between endothelial p16 expression, architectural growth patterns, and patient outcome in ccRCC.

### Cox regression analysis of overall survival

In univariable Cox regression analysis, high-risk architectural growth pattern was associated with significantly shorter overall survival compared with low-risk growth pattern (HR: 2.496, 95% CI: 1.597–3.899, *p* < 0.001). A high proportion of p16-positive vessels was associated with a reduced hazard of death compared with low vascular p16 positivity (HR: 0.462, 95% CI: 0.296–0.722, *p* < 0.001). High endothelial p16 staining intensity was also associated with a reduced hazard of death compared with absent staining (HR: 0.415, 95% CI: 0.211–0.815, *p* = 0.011). pT stage, tumor grade, nodal status, and distant metastases were additionally associated with overall survival in univariable analyses (Table 2).

**Table 2:** Cox regression analysis of overall survival: HR (hazard ratio); CI, confidence interval; LRP, low-risk pattern; HRP, high-risk pattern. Univariable Cox regression analyses were performed separately for each variable. The multivariable Cox regression model included pT stage, tumor grade, nodal status, distant metastases, architectural growth pattern, and the proportion of p16-positive vessels. The multivariable model included 199 cases and 76 events. Endothelial p16 staining intensity was not included in the multivariable model because the proportion of p16-positive vessels was selected as the primary vascular p16 variable

Variable	Comparison	Univariable Cox analysis *n*/events	Univariable HR (95% CI)	*p* value	Multivariable HR (95% CI)	*p* value
pT stage	pT3/4 vs. pT1/2	248/90	2.672 (1.758–4.063)	< 0.001	2.338 (1.425–3.835)	< 0.001
Tumor grade	G3/G4 vs. G1/G2	248/90	3.203 (2.016–5.089)	< 0.001	1.065 (0.560–2.026)	0.848
Nodal status	pN1 vs. pN0/cN0	244/90	3.541 (1.789–7.008)	< 0.001	1.109 (0.426–2.883)	0.833
Distant metastases	M1 vs. M0	244/90	3.471 (2.104–5.727)	< 0.001	2.919 (1.517–5.616)	0.001
Architectural growth pattern	HRP vs. LRP	215/79	2.496 (1.597–3.899)	< 0.001	2.070 (1.203–3.561)	0.009
p16-positive vessels	≥ 35% vs. < 35%	222/84	0.462 (0.296–0.722)	< 0.001	0.687 (0.404–1.169)	0.167
Endothelial p16 staining intensity	Score 1 vs. 0	222/84	1.036 (0.560–1.916)	0.910	—	—
Endothelial p16 staining intensity	Score 2 vs. 0	222/84	0.415 (0.211–0.815)	0.011	—	—

In the multivariable Cox regression model, which included 199 cases and 76 events, pT stage (HR: 2.338, 95% CI: 1.425–3.835, *p* < 0.001), distant metastases (HR: 2.919, 95% CI: 1.517–5.616, *p* = 0.001), and high-risk architectural growth pattern (HR: 2.070, 95% CI: 1.203–3.561, *p* = 0.009) remained independently associated with shorter overall survival. In contrast, tumor grade, nodal status, and the proportion of p16-positive vessels did not retain independent prognostic significance in the multivariable model (Table 2). The global Schoenfeld test did not indicate a violation of the proportional hazard assumption in the final multivariable Cox regression model (χ² = 7.19, df = 6, *p* = 0.304).

## Discussion

In this study, we demonstrate that endothelial p16 expression is a frequent finding in ccRCC and is associated with less aggressive clinicopathological features, favorable architectural growth patterns, and prolonged overall survival. In the multivariable Cox regression model, endothelial p16 expression was closely linked to architectural growth pattern and established clinicopathological parameters, but did not provide independent prognostic information beyond these variables. Thus, endothelial p16 expression appears to reflect a favorable vascular and morphologic tumor architecture associated with clinical outcome in ccRCC [10]. Clear cell RCC exhibits marked architectural heterogeneity, with individual tumors frequently displaying multiple growth patterns. Because our analysis was performed on tissue microarrays rather than whole-slide sections, assessment of architectural heterogeneity was limited to the sampled tumor areas and may not fully reflect the entire tumor architecture. Nevertheless, the small nest pattern represented the predominant growth pattern in our cohort, consistent with previous observations [10, 20]. To further evaluate the prognostic relevance of tumor architecture, we grouped thick trabecular/insular and solid sheet patterns as high-risk growth patterns, whereas all remaining architectures were classified as low-risk patterns. High-risk growth patterns were significantly associated with higher tumor grade and shorter overall survival, in line with previously published data [10].

The multivariable Cox regression model showed that HRP remained independently associated with shorter overall survival, whereas tumor grade did not retain independent prognostic significance. This may reflect a partial overlap between tumor grade and architectural growth patterns, since HRP were associated with less favorable tumor morphology and clinical outcome.

p16 is a central regulator of cell-cycle control and a widely used senescence-associated marker. Through inhibition of Cyclin D1/CDK4/6 signaling, p16 contributes to cell-cycle arrest and cellular senescence in response to various forms of cellular stress [21, 22]. Loss or downregulation of p16 expression has been described in a broad spectrum of malignancies [23], and ccRCC in particular has been reported to exhibit high rates of p16 negativity [24, 25]. Consistent with these observations, tumor cell p16 expression was infrequent in our cohort, with only 17.3% of cases showing focal p16-positive tumor cell clusters. Furthermore, tumor cell p16 expression was not associated with clinicopathological parameters, in line with previous findings by Maruschke et al., who likewise reported no association between p16 expression and tumor stage, grade, or metastatic status in ccRCC [26].

Interestingly, endothelial p16 expression was frequently observed in the intratumoral vasculature of ccRCC, with 87.7% of cases showing p16-positive blood vessels and a median of 35% positive vessels per tumor. The vascular nature of the examined cells was supported by corresponding CD31 immunohistochemistry. The immunoreactivity of p16 alone is not sufficient to establish functional senescence. Further studies with additional senescence-associated markers are still needed to clarify the biological nature of this p16 associated phenotype. Senescence-associated endothelial phenotypes have previously been described in a variety of non-neoplastic conditions, including chronic kidney disease, atherosclerosis, and diabetes mellitus [27–29]. In these settings, endothelial senescence-associated phenotypes have been linked to impaired vascular regeneration, altered metabolic activity, and inflammatory signaling [30, 31] Therapy-induced endothelial senescence has additionally been described following exposure to chemotherapeutic agents such as epirubicin and doxorubicin [32, 33].

In our cohort, reduced endothelial p16 expression was associated with higher tumor grade, advanced tumor stage, metastatic disease, and high-risk architectural growth patterns. Biologically, this may reflect a shift towards a more aggressive vascular tumor microenvironment in which vascular structures show less pronounced p16-associated features [13]. However, the mechanisms underlying reduced endothelial p16 expression in ccRCC remain unclear. Since p16 immunoreactivity alone does not establish functional cellular senescence, these findings should be interpreted as evidence of a vascular p16-associated phenotype rather than definitive endothelial senescence [34].

In contrast, comparatively little is known about senescence-associated endothelial alterations in neoplastic tissues. Previous studies reported p16-positive endothelial cells in benign vascular-rich tumors such as cardiac myxoma and hepatic hemangioma, where they were associated with inflammatory infiltration and increased vascular density [35, 36]. Data regarding malignant tumors remain scarce. To our knowledge, only isolated observations of endothelial p16 expression have been reported in melanoma brain metastases [37], whereas senescence-associated vascular phenotypes were linked to adverse prognosis in chondrosarcoma and glioblastoma [38, 39].

In the present study, endothelial p16 expression was strongly associated with favorable clinicopathological characteristics, including lower tumor grade, lower tumor stage, absence of metastases, and prolonged overall survival. In addition, increased endothelial p16 expression correlated with distinct architectural growth patterns, as high-risk growth patterns exhibited lower endothelial p16 positivity compared with low-risk patterns. Interestingly, stratified survival analyses demonstrated that the prognostic impact of architectural growth patterns differed according to the endothelial p16 expression. High-risk growth patterns were associated with significantly shorter overall survival only in tumors with low endothelial p16 positivity, whereas no significant survival differences between architectural subgroups were observed in tumors with high endothelial p16 expression. These findings support a close relationship between vascular phenotype and tumor architecture in ccRCC.

Senescent cells undergo stable cell-cycle arrest but remain metabolically active and secrete a broad range of bioactive molecules collectively referred to as the senescence-associated secretory phenotype (SASP). The SASP includes pro-inflammatory cytokines, growth factors, chemokines, and matrix-remodeling enzymes that can influence the surrounding tissue microenvironment [40]. Depending on the biological context, senescence-associated phenotypes may exert both tumor-suppressive and tumor-promoting effects, particularly in tissues exposed to chronic cellular stress or premature senescence [41].

In our cohort, increased endothelial p16 expression was associated with less aggressive tumor features and prolonged overall survival, suggesting that senescence-associated endothelial phenotypes may be linked to a biologically less aggressive vascular microenvironment in ccRCC. This observation contrasts with findings in chondrosarcoma and glioblastoma, where vascular senescence-associated phenotypes were linked to adverse clinical outcome [42, 43]. One possible explanation may be that endothelial p16 expression in ccRCC occurs preferentially in lower-stage tumors, where senescence-associated mechanisms could still contribute to tumor-suppressive barrier functions.

It is also conceivable that endothelial p16-positive cells in ccRCC do not represent fully senescent cells but rather a pre-senescent endothelial phenotype lacking the pronounced pro-inflammatory secretory profile typically associated with terminal senescence [44]. This interpretation may also be consistent with the observation that lower levels of intratumoral immune cell infiltration are associated with favorable prognosis in ccRCC [45].

Collectively, our findings support the concept that the vasculature of ccRCC is biologically heterogeneous and that endothelial p16 expression identifies distinct vascular phenotypes associated with tumor morphology and clinical outcome. This study is limited by its retrospective single center design and the lack of an independent external validation cohort, Further studies are required to clarify the functional significance of endothelial p16 expression and its relationship to vascular senescence in ccRCC.

## Conclusion

We showed that p16 expression is more commonly observed in endothelial cells of ccRCC rather than in tumor cells themselves. Vascular p16 expression is associated with low-risk growth patterns as well as a longer overall survival time. These findings suggest that p16 expression in tumor associated endothelial cells reflects a more favorable vascular and architectural tumor growth together with clinical outcome.

## Data Availability

Datasets used and analyzed in this study are available from the corresponding author on reasonable request.

## Abbreviations

ccRCC: clear cell renal cell carcinoma
FFPE: formalin-fixed paraffin-embedded
H&E: hematoxylin and eosin
HIF: hypoxia-inducible factor
HRP: high-risk pattern
IHC: immunohistochemistry
ISUP: International Society of Urological Pathology
LRP: low-risk pattern
RCC: renal cell carcinoma
SASP: senescence-associated secretory phenotype
TMA: tissue microarray
TNM: tumor-node-metastasis
VEGF: vascular endothelial growth factor
VHL: von Hippel-Lindau
